# A Case of Femoral Fracture in Klippel Trenaunay Syndrome

**DOI:** 10.1155/2014/548161

**Published:** 2014-11-12

**Authors:** Sam Nahas, Fabian Wong, Diane Back

**Affiliations:** Guy's and St Thomas' NHS Foundation Trust, St Thomas' Hospital, Trauma Room, Ground Floor, Westminster Bridge Road, London SE1 7EH, UK

## Abstract

We present a case of Klippel Trenaunay syndrome (KTS) who presented with severe bilateral knee osteoarthritis (OA). Preoperative planning was commenced for a total knee replacement (TKR). Whilst on the waiting list the patient suffered a fall and sustained a complete femoral diaphysis fracture. Conservative management in the form of skin traction was initially chosen as significant extra- and intramedullary vascular malformations posed an increased risk of perioperative bleeding. This failed to progress to union, and so open reduction and internal fixation was performed. This subsequently resulted in on-going delayed union, which was subsequently managed with low intensity pulsed ultrasound (LIPUS, otherwise known as Exogen (Bioventus. exogen. Secondary exogen, 2012)). There are only two previous documented cases of femoral fracture in KTS. This is the first report of a patient with this rare syndrome receiving this treatment. We discuss the management of fracture in this challenging group of patients.

## 1. Background

Klippel Trenaunay syndrome is a rare congenital condition characterised by a triad of (a) cutaneous naevi (capillary malformations—the “port wine stain”); (b) varicose veins or venous malformations; and (c) hypertrophy of bones and soft tissues of the extremity [1]. 70% of cases present in the lower limb and 11% in the upper limb [2]. It is one of at least 9 vascular malformation syndromes that can affect the limbs [3]. The condition commonly presents itself with pain in the lower limb, bleeding, functional limitation, and cosmetic sequelae as a result of venous malformation or tissue overgrowth. The prevalence of the condition is 1 in 20–40,000 [4] and although the aetiology is unknown, there are multiple theories [2]. One of which is that there is an error in mesodermal proliferation in utero [1].

If operating on the limb, the pathology of this condition can prove a very challenging management scenario. Venous malformations have been reported to bleed excessively during orthopaedic procedures, requiring copious blood transfusion. With regards to orthopaedic operations it is also known that patients with KTS can have osteoporotic bone. This can prove difficult when making decisions regarding the most appropriate management of any elective procedure or traumatic bony injury.

We describe a case of a patient with KTS whom sustained a complete femoral diaphysis fracture. Operation was avoided, but became inevitable due to nonunion. This meant open reduction and internal fixation had to be performed in addition to LIPUS.

There have only been two previous reported cases [5, 6] of fracture in this challenging group of patients. Therefore further experience needs to be collated in order to provide a better evidence-based approach of management.

## 2. Case Presentation

**Figure 1:**
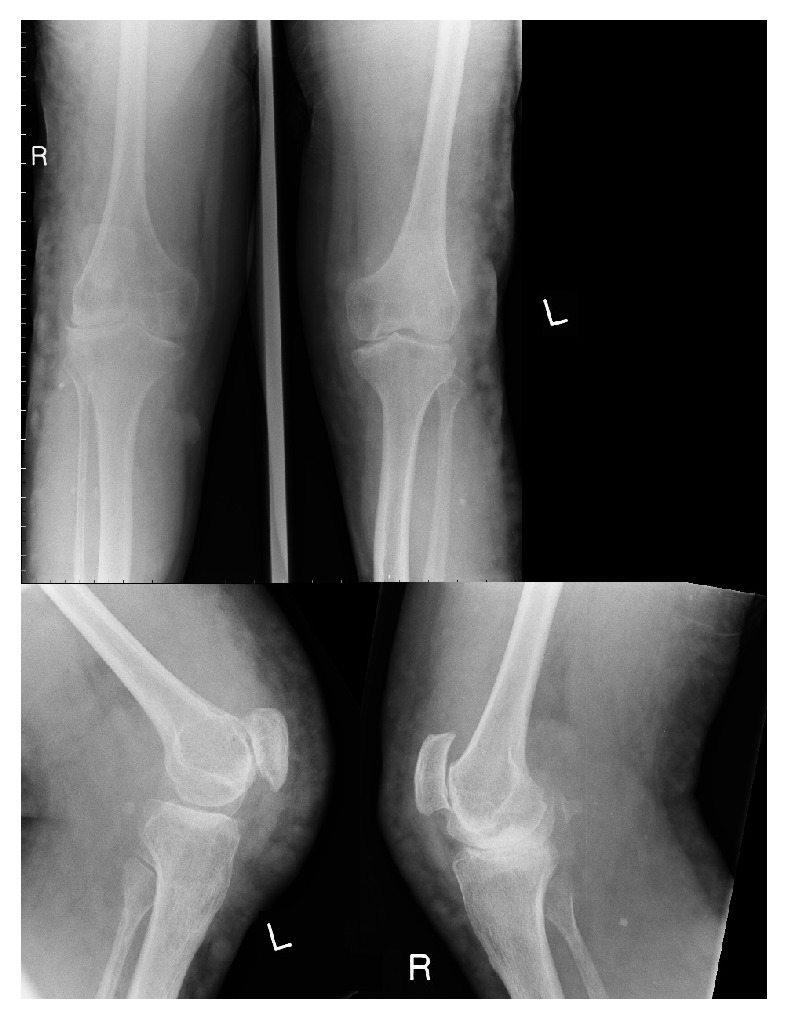
AP and lateral radiographs of the patient's knees at age 21.

This gentleman presented initially to the orthopaedic clinic at the age of 21. His main complaint was severe bilateral knee pain, which had been long standing. He had multiple hemarthroses of both knees as a child, which were the result of recurrent falls. He also had a history of the left tibia fracture aged 9 that healed with malunion. On examination the left tibia was bowed and there were extensive cutaneous venous malformations over both legs posteriorly, laterally, anteriorly, and extending above both knees. Plain radiographs were performed and confirmed tricompartmental osteoarthritis of both knees (Figure 1). In view of the peculiar history of hemarthroses and venous malformations he was at this stage referred to the vascular specialists. Bilateral whole leg MRI scans were performed with short TI inversion recovery (STIR) sequence. This demonstrated a “classical vein of Servelle” and extensive vascular malformation involving buttocks and legs bilaterally, confirming a diagnosis of Klippel Trenaunay syndrome. The vein of Servelle is a pathognomic finding and is a pathological vein found in the lateral calf and thigh [7].

At this stage the patient was encouraged to undertake conservative measures in order to manage the symptoms of osteoarthritis. He attended the outpatient clinic for intermittent review over several years. At age 23 his pain became more intense, being significantly worse in the right knee. He had at this point also developed a fixed flexion deformity of this knee. Repeat MRI showed gross degeneration particularly within the patellofemoral joint. It was thought prudent at this stage that some form of intervention would be required due to failure of conservative measures. He then had an uneventful bilateral knee arthroscopy, synovectomy, and debridement. This improved the symptoms dramatically in the right knee, but not the left.

He continued to be seen regularly in clinic for the following nine years before he presented with the increasing inability to cope, uncontrollable pain, and night pain. On examination the right knee had a very poor range of motion—being in 25-degree fixed flexion to a maximum of 35 degrees extension. Knee replacement of the right knee was indicated at this stage and presurgical planning was arranged. Computer tomography (CT) and a repeat MRI were performed, confirming the pathological anatomy of the tibia as well as the presence of extensive intra- and extramedullary venous malformations.

**Figure 2:**
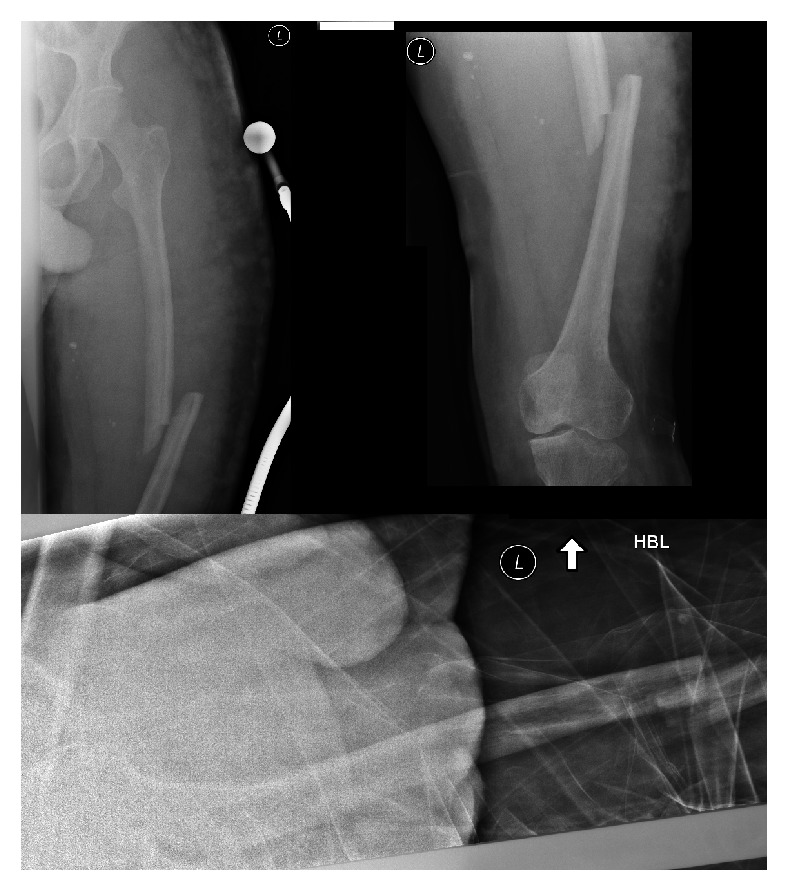
AP and lateral radiographs of the left femur, demonstrating a complete diaphyseal fracture.

The patient was on the waiting list for the knee replacement (age 29) when he fell at home in his kitchen onto his left leg and sustained a closed, displaced, oblique midshaft diaphysis fracture of the femur (Figure 2). This was an isolated injury.

Following advice from the vascular surgeons and review of current literature, it was decided that nonoperative management should be attempted owing to bleeding risk. He was initially treated in skin traction. Reluctantly and after some deliberation, a decision was made that skin traction was ineffective and therefore a tibial pin was inserted to allow skeletal traction for the subsequent 4 weeks. At the end of this period, clinical examination revealed ongoing mobility at the fracture site, while plain radiographs were obtained and confirmed nonunion. For this reason ORIF was performed.

**Figure 3:**
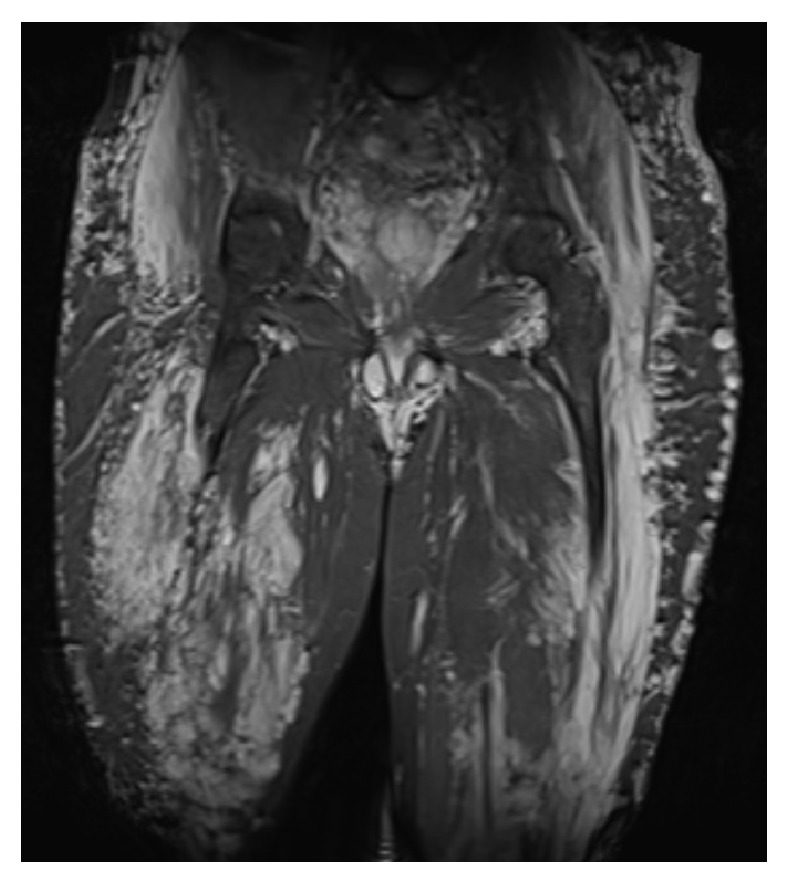
Coronal MRI STIR sequence of the anterior thigh.

**Figure 4:**
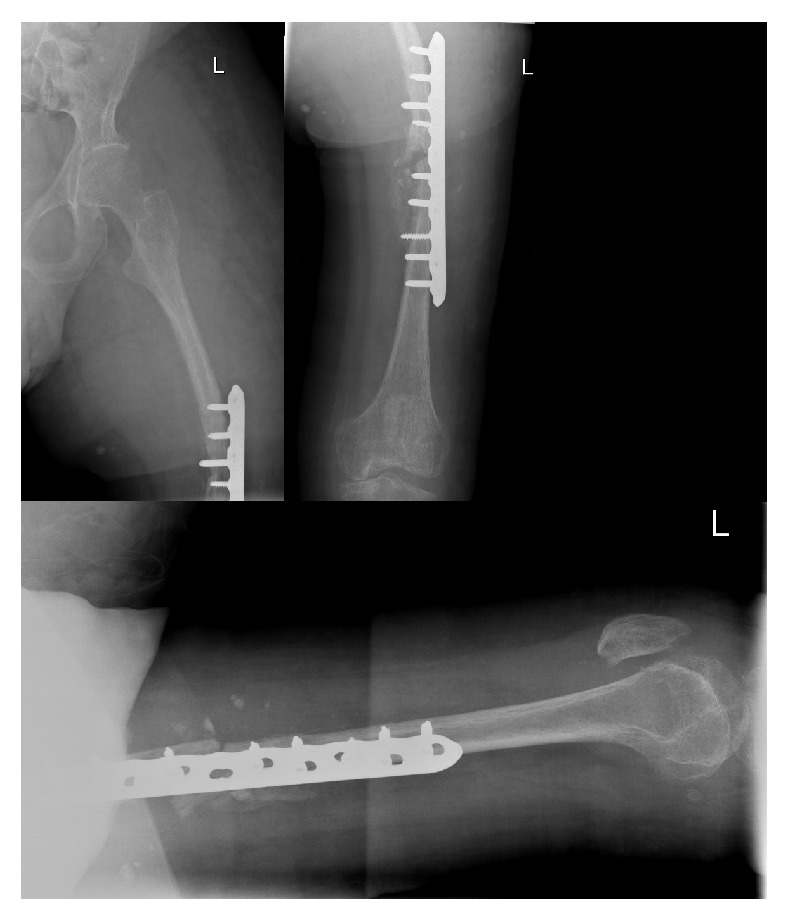
AP and lateral radiograph of the left femur immediately after open reduction, internal fixation.

During the operation he required 15 units of blood due to excessive bleeding from the venous malformations. A tourniquet was applied for a total of 115 minutes. A vastus lateralis splitting approach was taken in order to reduce tension on surrounding structures. It was noted that the venous malformations were abundant in the whole upper leg region (which can be seen on the MRI scan, Figure 3). Bone quality was noted to be poor. A 10-hole locking compression plate with 4 proximal and 5 distal locking screws was used (Figure 4). The locking plate was chosen over a femoral nail due to the extensive vasculature within the medullary canal (Figure 3). Vasculature laterally appeared less widespread. All layers were closed with Vicryl.

**Figure 5:**
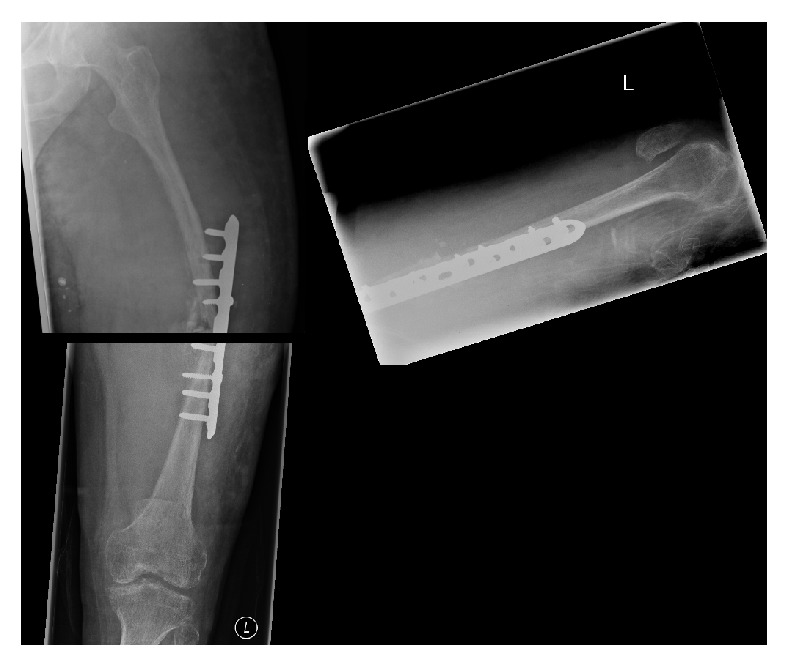
AP and lateral radiograph of the left femur immediately one month after open reduction, internal fixation.

Radiographs one month after the ORIF showed no evidence of callus formation (Figure 5). At this stage there is evidence of loosening possibly due to poor bone quality. The patient expressed a wish at this point to consider amputation but was also willing to consider alternative options. Following further literature review for the management of nonunion and after discussion with patient, he was commenced on LIPUS [8] for 20 minutes daily.

**Figure 6:**
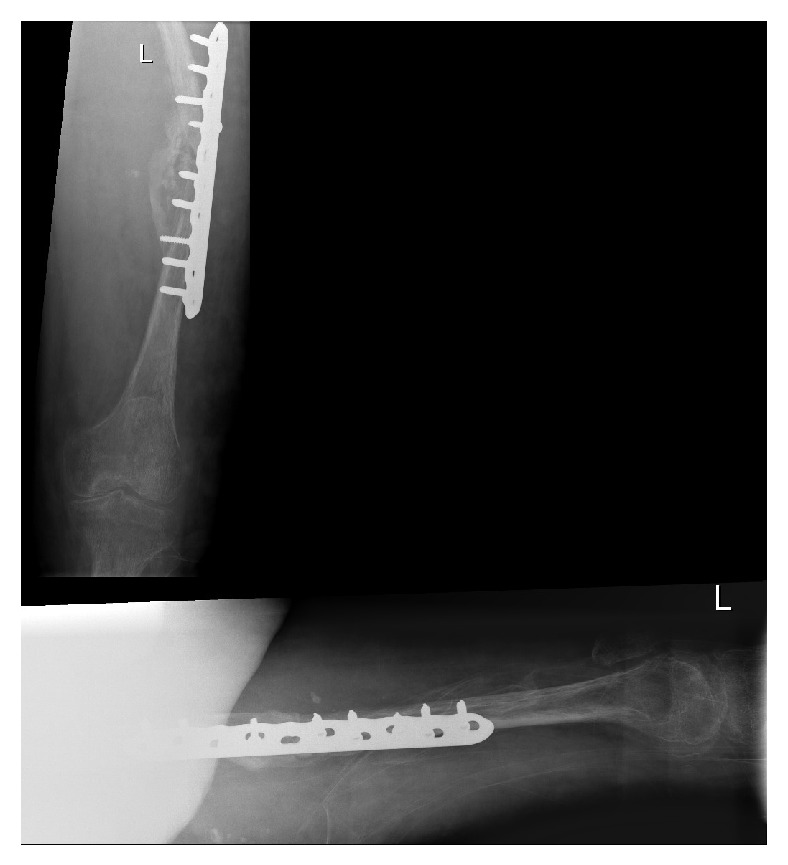
AP and lateral radiograph of the left femur after 10 weeks of LIPUS therapy.

There was limited evidence for clinical or radiological union after initial four weeks of LIPUS; however it was agreed that it was worth trying LIPUS for further six weeks, given there was no formal guideline on duration of treatment from Exogen [8] and that some studies have shown evidence of beneficial effect after using LIPUS for 20 weeks [9]. After a total of ten weeks with LIPUS treatment, there was clinical evidence of union with radiological evidence of malunion (Figure 6). Hydrotherapy and gentle physiotherapy were commenced and discharge from hospital was arranged. He attended outpatient follow-up on a regular basis with subsequent radiographs continuing to show callus formation. Since writing the report he has been lost to follow-up.

## 3. Discussion

We report a 32-year-old man with KTS who suffered a complete diaphyseal fracture of the left femur. This is a condition in which there are vascular and bony abnormalities—which can cause challenging management dilemmas in orthopaedic surgery.

There are only two other reported cases of fracture in KTS. Tsaridis et al. [5] were the first to describe their encounter with a very similar injury. An intramedullary nail was used to achieve internal fixation. This patient eventually made a full recovery. The second case as reported by Notarnicola et al. [6] illustrated another very similar fracture but the recovery was overshadowed by severe disabling neuropathic pain. This patient also showed complete union of the fracture one year after the accident.

Both of the aforementioned cases were similar injuries of the femur, with the patients in these cases being 42 and 52 years of age. The former occurred as a result of a road traffic accident and the latter an “accident fall at work.” Our patient fell from standing, which would indicate that it was a fragility fracture. Although the energy of force is not clear from the other case reports, it may be possible that KTS affects bone remodelling and therefore bone quality. Notarnicola et al. reported very poor bone quality in their patient, with a patchy and mottled diaphyseal cortex similar to that found in our patient [6]. This osteoporotic effect was also described by Redondo et al., who recommended prophylactic bisphosphonates in patients with the condition [3].

Servelle [10] operated on a total of 728 patients with KTS. He demonstrated that 36% had clinically manifest varicose veins, and 32% had flat angiomata. It is clear from the previous reports that the degree of venous malformation is extremely variable in different areas of the limb. Despite our best efforts, the patient in question bled extensively during operation—requiring 15 units of blood on the operating table, even with an emphasis to optimise surgical wound size and to achieve haemostasis. Tsaridis et al. had a similar problem despite using a minimally invasive approach with the insertion of a femoral intramedullary nail. Notarnicola et al. had less of an issue with intraoperative bleeding, although still requiring 4 units, despite the apparent same technique as reported by Tsaridis et al. It is impossible to compare these cases directly, however, as the extent of the vascular malformation in the other cases is unknown as MRI scans were not performed [3]. The other authors also did not describe their approach in detail.

With vascular malformation in mind, the route of any orthopaedic procedure on patients with KTS needs to be considered strongly in both trauma and elective cases. Minimally invasive surgery should be attempted where possible. Conservative measures such as casting or traction can be considered. Owing to the slow healing rate in this group of patients, conservative management must be weighed carefully with increased risk of bleeding in operative management. We have demonstrated that despite extensive imaging of vasculature, haemostatic control is very difficult. Despite this, imaging is recommended none the less, as atypical large veins are seen in 72% [2].

Articular involvement, as in our patient's case, has been previously documented in subjects with extensive vascular malformation of the lower limb [3]. Our patient suffered from bilateral recurrent traumatic haemarthrosis as a child. Osteoarthritis can occur early as a result of a chronic synovial inflammatory response to intra-articular bleeding.

Our patient suffered from delayed bone healing. It has been suggested that slow bone healing is a common feature of vascular malformation syndromes [3]. With possible partial success we have illustrated that the use of LIPUS, although not trialled in this patient group, has shown possible evidence of benefit. LIPUS has shown strong evidence that it can stimulate bone healing in fresh fractures. Overall, literature shows that healing rates of fracture nonunion, however, are between 73 and 90% if LIPUS is used in the normal population and without the need to reoperate [11]. This is dependent on multiple factors, however, such as the site and how quickly after the original operation it is used. It has been shown experimentally to increase callus tissue and accelerate bone consolidation. Several studies have shown it is beneficial in the use of lower limb fracture nonunion if surgical intervention has failed to consolidate [12, 13]. This has the advantage of avoiding the need for further corrective operation on the fracture if union does not occur in the first instance. In our case, this had the upmost importance, as reoperation could be fatal.

In conclusion, managing femoral fracture in patients with KTS is an extremely complex issue and needs careful operative planning. Main operative complications include extensive haemorrhage and delayed bone healing. These issues can be addressed in the first instance by prescribing prophylactic bisphosphonates as per secondary prevention. Having MRI imaging preoperatively in order to approach planning can reduce risk of bleeding. With reference to delayed bone healing LIPUS can be considered postoperatively. The risk of fracture site movement in conservative management due to slow bone healing should be carefully balanced against bleeding risk in open reduction and internal fixation.

## 4. Learning Points/Take Home Messages

- In managing any fracture despite the pathology basic principles should always be adhered to.
- MRI STIR imaging is essential in patients with vascular abnormality syndromes undergoing surgery.
- Low intensity pulsed ultrasound is potentially a useful tool in treating nonunion in vascular disorders such as KTS.
